# Toward a framework of moral challenges in military operations: implications for psychological resilience

**DOI:** 10.3389/fpubh.2026.1813972

**Published:** 2026-08-12

**Authors:** Megan M. Thompson, Joshua A. Granek, Oshin Vartanian

**Affiliations:** 1 Influence, Intelligence, and Collaboration Section, Toronto Research Centre, Defence Research and Development Canada, Toronto, ON, Canada; 2 Human Effectiveness Section, Toronto Research Centre, Defence Research and Development Canada, Toronto, ON, Canada

**Keywords:** extended reality (XR), fMRI, military personnel, moral challenges, moral decision-making, moral injury, potentially morally injurious events (PMIES)

## Abstract

This perspectives paper presents a programmatic integrative review and preliminary framework informed by a multi-year, multi-method research program conducted at Defence Research and Development Canada – Toronto Research Centre to document moral experience in military operational contexts. Drawing on a multidisciplinary, empirically grounded program integrating qualitative, experimental, field, and survey methods, the paper outlines a framework in which operational environments can give rise to moral challenges—ethically salient situations that may test values or the ability to act—which can escalate into moral conflicts, where values compete, and in some cases moral dilemmas, where competing obligations are irreconcilable. Engagement with these situations involves interacting affective, cognitive, physiological, and identity-related systems and may generate moral stress, defined as psychological strain associated with prioritizing competing values under conditions of high consequence and constrained agency, even in the absence of wrongdoing. These processes may lead either to moral resolution, characterized by integration and meaning-making, or, if unresolved, to moral distress, moral injury, and other negative psychological outcomes. Integrating findings across methods, this perspective advances understanding of psychological resilience through a moral lens, with potential applications to first responders who similarly confront morally challenging situations.

## 1 Introduction

### 1.1 Conceptual challenges of a multidisciplinary approach

A multidisciplinary approach to the moral domain is inherently challenging because philosophical, spiritual/existential, psychological, healthcare, and military traditions have largely evolved as separate bodies of knowledge, with limited integration across disciplines. This fragmentation, widely noted in the literature, has led to limited conceptual coherence, ambiguity in definitions, and inconsistent terminology across fields. For example, constructs such as moral distress and moral stress show both overlap and inconsistent application (1, 2), while reviews of moral injury (MI) highlight ongoing debate regarding definitions, boundaries, and measurement (3–6). Consequently, terms including moral challenge, demand, conflict, judgment, reasoning, stress, distress, and injury are variably defined, inconsistently applied, or unevenly represented across disciplines. This lack of coherence complicates efforts to establish a unified language of moral experience. While resolving these issues is beyond the scope of this work, Table 1 provides a pragmatic summary of the constructs and definitions used herein to guide the reader.

**Table 1:** List of moral concepts and definitions.

Deployment: a longer assignment where a unit is sent to a location (often overseas or in a crisis area) for a set period of time.
Mission: a single task or operation with a specific objective (e.g., patrol an area, deliver supplies, conduct a raid).
Moral situation: a situation that implicates values, affects others, or can be evaluated as right/wrong, including routine decisions and low-stakes interactions.
Moral challenge: any moral situation that may test a person’s values or ability to act, but can still be resolvable.
Moral conflict: when two or more moral obligations or demands contradict. Competing values may or may not be resolvable; where one value can sometimes be judged as more important or overriding, and a clear “right” answer may exist.
Moral dilemma: a specific type of moral conflict where two or more important moral values or obligations are mutually incompatible and cannot all be satisfied, and no option is clearly superior; a loose-loose situation.
Potentially morally injurious events (PMIE): a special subset of moral challenge involving witnessing, committing or failing to prevent harm to others and/or betrayal that has the potential for the heightened severity and consequences associated with moral injury.
Moral intensity: features of the moral situation that influence the moral urgency of the situation, affecting awareness, judgment and behavior. These include magnitude of consequences, temporal immediacy, proximity, social consensus, concentration of effect.
Moral awareness: the ability to recognize that situation has moral significance.
Moral reasoning: the cognitive process by which individuals evaluate situations apply moral principles, values, or norms, and weigh priorities in order to determine what is right or wrong.
Moral judgment: the conclusion that a person makes about whether an action is right or wrong.
Moral intent: the commitment to act based on one’s moral judgment.
Moral competence: the capacity to effectively apply moral awareness, reasoning and action.
Moral emotions: a subset of affective responses that are directly linked to moral values, judgments, and concerns about right and wrong, and that influence how individuals evaluate, respond to, and act in ethical situations. (1) Directed at: Self (guilt, shame, pride) (2) Other’s violations (anger, disgust, contempt); (3) Others suffering (empathy, sympathy, compassion)
Conscience: a self-evaluation and regulatory system, closely tied to moral emotions like guilt shame, pride, which guide behavior.
Honor: conceptualized as a military profession identity -specific form of conscience—as well as contempt, anger, compassion, and distress at others’ suffering.
Moral stress: the general psychological strain from associated with recognizing a moral demand or conflict, even if they are still able to act according to their values.
Moral resilience: the capacity of an individual to sustain, resolve or restore the coherence of their moral identity in response to moral complexity, conflict or dilemma.
Moral distress: arises when a person knows the ethically appropriate action to take but is unable to act on it due to external constraints (e.g., institutional rules, authority, lack of resources).
Moral injury: severe and lasting psychological harm related to a moral violation or transgression.

### 1.2 Psychological resilience

Although psychological resilience has traditionally been defined in terms of stress adaptation and recovery (7), like others we advocate for the inclusion of and a deeper understanding of the moral domain as a crucial component of its phenomenology. For instance, Rushton (8) defined moral resilience, specifically as it relates to the health care profession, as the capacity of an individual to sustain or restore their integrity in response to moral complexity, confusion, distress or setbacks. Although scientific attention to the moral domain is increasing (6, 9, 10), moral challenges, i.e., those situations involving uncertain or competing morally relevant values, obligations or principles (11–14) and potentially morally injurious events (PMIEs), i.e., a type of intense and consequential moral challenge involving committing or witnessing a moral transgression that violates deeply held moral beliefs or expectations (4, 14) have long been recognized among first responders (15, 16), including military personnel (17–19).

Research increasingly documents the frequency with which military personnel and first responders encounter moral challenges and potentially morally injurious events (PMIEs), as well as their associated consequences, including moral distress and moral injury, with implications for psychological resilience. This work has identified the more common sources of moral challenge in military contexts, where tensions often arise among rules of engagement, operational realities, and personal or cultural values, such as constraints on intervening in civilian suffering. Contextual and organizational factors—including uncertainty, time pressure, danger, emotional intensity, and ethical climate—have been cited as shaping exposure to and responses to these challenges (e.g., 19–24, 69). Although this literature has been very informative, there have been growing calls for greater methodological integration and interdisciplinary approaches capable of capturing the dynamic interplay among cognition, emotion, and environment in morally challenging situations (25–27). In response, the present work integrates multiple strands of research to advance a set of initial propositions regarding the moral challenges of military operations, with broader implications for understanding psychological resilience among first responders who also routinely confront moral challenges in their occupations.

## 2 An integrated approach to documenting moral challenges, moral decision-making processes and moral distress/injury in military operations

### 2.1 Interview studies

In the mid-1990s, the Toronto Research Centre (TRC) research program’s initial study examined the experiences of Canadian Armed Forces (CAF) military augmentees^2^ and revealed that even operations portrayed as a relatively low-risk peacekeeping deployment in Bosnia could include profound moral challenges and potentially morally injurious experiences. These poignant accounts underscored the psychological and spiritual/existential toll of witnessing atrocities, often while being unable to intervene, despite having the training and capacity to do so, as illustrated by this soldier’s account:

*Nobody ever trained you to sit in a compound and watch women and children being shot like animals … you could do nothing about it … one of my friends could not live with himself … watching all these women and children being butchered and he could do nothing … That’s got to be the worst thing that a human being can do. There you are, trained, you have got weapons in your hand, yet there’s absolutely nothing you can do.* — CAF soldier, Thompson & Gignac (28).

This quote illustrates how potentially morally injurious events can occur even in deployments involving low personal safety threat and begins to speak to the experience of moral distress, which Jameton (29) defined as the psychological pressure that arises when a person knows the ethically appropriate action to take but is unable to act on it due to external constraints (e.g., institutional rules, authority, lack of resources) and moral injury, the severe and lasting psychological harm related to perpetrating or witnessing a moral violation or transgression, or experiencing betrayal (2). Statements such as these provided the foundation for what would become a multi-year -method research program examining the moral realities of military operations.

Our next study delved into these issues further. Qualitative interviews with fifteen serving or recently retired senior CAF commanders documented thirty-three morally complex operational dilemmas (30, 31). Using an integrated analytic framework drawing on moral decision-making and moral distress/injury literatures, we examined situational context, judgment processes, and indicators of unresolved moral stress, including statements referring to moral emotions of guilt, shame, and/or indicating a lack of cognitive or emotional resolution, i.e., that the person has not fully processed, integrated, or settled their thoughts and feelings about a moral challenge.

Results indicated that the most common moral conflicts^3^ recounted involved competing obligations, primarily conflicts between rules of engagement and operational realities (32). Key moral intensity dimensions (33) mentioned included magnitude of consequences, proximity, probability of effect, and temporal immediacy. Other moral emotions included conscience, i.e., an internalization of standards for self-evaluation and -regulatory system and closely tied to moral emotions like guilt shame, pride, which guide behavior (see (34)) and honor—which we conceptualized as a military profession identity -specific form of conscience—as well as contempt, anger, compassion, and distress at others’ suffering (35). Seven accounts were coded as unresolved, reflecting an ongoing degree of moral stress characterized by greater proximity, more severe consequences, and heightened moral emotions. Narratives revealed the interplay of personal values and identity (see (3)) with contextual constraints. The accounts tended to describe initial intuitive, emotionally driven judgments giving way to more deliberative reasoning, persistent perceptions of institutional constraint, and challenges to post-event identity coherence.

This study provides a relatively rare systematic examination of how senior military leaders experience, process and live with morally complex operational decisions. Integrating qualitative interviews with moral decision-making theory, moral intensity dimensions, and a preliminary assessment of psychological resolution, it suggests that competing obligations, i.e., moral conflict —particularly tensions between rules of engagement and operational realities—interact with moral emotions and institutional constraints to shape decision making with links to moral stress and moral distress. Moreover, in attempting to distinguish resolved from unresolved accounts, the study points to potential differences in post-event trajectories, raising the possibility that psychological resilience, in this context, may be at least partly involve processes associated with identity coherence, meaning-making, and living with the consequences of constrained moral judgment.

### 2.2 Experimental studies

We next adapted two commander narratives into controlled experimental scenarios to examine patterns in moral decision making and differences between civilian and military respondents (36–38). Participants assumed the role of a deployed commander confronting two dilemmas: whether to admit civilians fleeing shelling into a military encampment, and whether to privately reprimand or court-martial a trusted subordinate engaging in risky, unsanctioned behavior – both implicating the balancing of humanitarian motivations against operational neutrality. Civilians about were equally divided on refugee entry, whereas about 80% of military participants denied entry, while the subordinate discipline decisions were more evenly distributed across groups.

The results also suggest the perception of a degree of moral conflict. Participants rated care-based concerns at levels comparable to consequence- and role-based considerations (37). Similarly, although denying entry to refugees was judged less just and less socially endorsed, it was also perceived as producing less overall harm (36, 38). Military participants tended to justify mission-aligned choices (e.g., refusing entry to the refugees) through broader deployment-level and long-term considerations rather than solely rule compliance. These results offer additional insights into moral decision-making in operationally framed contexts involving competing considerations under uncertainty, and suggest that the observed differences may reflect factors such as role requirements and training.

### 2.3 Field studies

Although scenario-based training is central to military preparation (39), its effectiveness for ethical decision making remains under-researched (40–42). In one notable exception Warner et al. (43) demonstrated that mid-deployment ethics training using vignettes and leader-led discussion reduced self-reported unethical behavior and improved clarity regarding conduct toward non-combatants. Addressing this issue, the TRC research team conducted field studies examining how military personnel make moral decisions during ethically complex operational training scenarios. This approach allowed for the systematic manipulation of factors during ethically complex, high stress training settings. As a result, moral decision-making could be observed as it unfolded under conditions approximating operational pressure, while still allowing for the documentation of decision points and decision-making processes and comparison of responses across standardized scenarios.

This work was embedded in a scenario in the final field phase of the United Nations Military Observer (UNMO) Course at the Canadian Armed Forces Peace Support Training Centre, where reservists acted as role players in a scripted human rights violation scenario involving civilians abused by local militia during a day-long exercise. Because UNMOs are unarmed, the course emphasizes information gathering, negotiation, and personal and mission safety in potentially volatile environments. Volunteer trainees were informed at the start of the day that the research focused on operational decision making and that video recording and decision-making questionnaires would be used; informed consent was obtained. To preserve naturalistic behavior, trainees were not told which scenario was being studied or that moral choices, attitudes and behaviors were the focus. Research personnel, cameras, and questionnaires were introduced only after the targeted scenario had concluded.

One study experimentally manipulated the proximity of victims (44), demonstrating that closer victim proximity heightened trainees’ self-reported moral commitment to saving lives but did not increase confrontational behavior. Trainees also evaluated themselves more critically when unable to save victims despite well-intentioned efforts. A second study (31) manipulated the anger level of the militia leader to test predictions derived from social contagion (45) and strategic choice (46) theories. Relative to the neutral condition, when confronted with an angry leader, trainees intensified their de-escalation efforts, demonstrated greater responsiveness and compliance, and reported heightened empathy, while simultaneously expressing more negative attitudes toward the militia. However, trainees’ behavior was also more frequently rated by coders as condescending, insulting, or threatening.

Together, these studies provide relatively rare behavioral data collected in high-fidelity training environments designed to approximate operational conditions. By embedding controlled manipulations—such as victim proximity and the emotional volatility of an armed actor, they offer preliminary evidence of the influence of contextual factors on moral decision-making. Specifically, these factors may shape not only behavioral responses but also moral self-assessments and self-regulation—processes identified in prior literature as relevant to psychological resilience.

### 2.4 Survey research

An early review identified MI as a significant psychological sequela of military operations and called for greater prioritization within CAF health policy (47), a position later supported by a large-scale survey showing that over half of a sample of approximately 5,000 CAF personnel reported exposure to potentially morally injurious experiences (PMIEs) during their deployments, and that such exposure was associated with a significantly greater likelihood of past-year PTSD and major depressive disorder (MDD) compared to those not PMIE-exposed in operations (48). Related findings extend this pattern to non-traditional operations: CAF personnel deployed into long term care facilities (LTCFs) during the crisis phase of the COVID-19 pandemic also reported exposure to a range of morally challenging conditions alongside elevated levels of moral emotions (shame and guilt) as well as moral distress, moral injury, depression, and PTSD symptoms (49). Together, these studies provide evidence that PMIES may be relatively common deployment experiences, and tend to co-occur with both moral emotions (e.g., guilt, shame) and adverse mental health outcomes following morally challenging experiences. These findings continue to speak to the potential value of extending psychological resilience models to include a moral dimension.

### 2.5 International collaborations

Complementing this work, participation in NATO Research Task Groups (‘Moral Decisions and Military Mental Health’ and ‘Moral Challenges in the Future Security Environment: Guidance for Leaders’) has begun to highlight the cross-national relevance of moral challenges in contemporary military operations (50) and helped to identify practical priorities. These include embedding moral scenarios in training (41, 43) fostering ethically engaged leadership (51–53), and strengthening integrated moral, spiritual, and psychological supports (54). Collectively, these efforts suggest that moral challenges can be often present in military operations, are often linked to negative psychological outcomes and may be affected, in part, through training, leadership, and institutional support.

### 2.6 Using extended reality to prepare for moral complexity in operations

Our prior work has pointed to the emerging need to determine how best to prepare personnel for the moral situations they may encounter in order to support sound decision-making and mitigate associated psychological impacts. Traditional military ethics education has relied primarily on classroom-based instruction, which remains essential for conveying core values and norms but may offer limited exposure to the complexity, uncertainty, and emotional intensity of operational moral dilemmas (42, 55). Although embedding moral challenge scenarios in live training has been recommended, the significant time and resource demands of such approaches can constrain their widespread implementation (41), motivating the exploration of alternative methods within this research program.

Emerging technologies such as extended reality (XR) offer a promising approach for preparing military personnel for moral challenges and potentially morally injurious experiences by enabling more embodied engagement with ethically complex situations, complementing abstract moral reasoning. Emerging evidence suggests that immersive training may enhance moral awareness and decision making under ambiguity, influence moral reasoning, and support the regulation of physiological stress responses (56–58). Building on this work, we are leveraging a multimodal immersive testbed to develop and assess operationally relevant moral challenges in real time incorporating moral decision making, behavioral, and physiological indicators, to assess their potential as a training approach and to explore whether such systems can be used to identify early indicators of moral strain, which may begin to contribute to understanding when it begins, how it unfolds, and which individuals show heightened sensitivity or adaptive coping.

As an emerging area, several important issues require further investigation. The protective effects of XR remain uncertain, and questions of transfer, durability, generalizability, and potential adverse effects underscore the need for careful design, co-development with military stakeholders, and integration within a broader ethical and mental-health training ecosystem.

### 2.7 Neural correlates of moral injury

A further area of growing interest is the neuroscience of MI, which reflects the promise of identifying brain systems associated with MI to clarify underlying psychological mechanisms (59). Because neuroimaging is correlational (60), such findings must be integrated with developmental, behavioral, and clinical data. Below is short review of the emerging, albeit still small body of research shaping a future direction in our research program. We divide that literature into the specific paradigms involved and the inferences that can be drawn from them.

Resting-state functional magnetic resonance imaging (rs-fMRI) studies—i.e., scans acquired while participants are awake but not engaged in any specific task—have been used to assess intrinsic neural activity and functional connectivity. Findings suggest that MI-related experiences are associated with differences in regions of the default mode network (DMN), particularly those involved in self-referential processing and autobiographical memory. Sun et al. (61) found that amplitude fluctuations in the left inferior parietal lobule correlated positively with moral-injury transgressions and negatively with betrayals (assessed via the Moral Injury Event Scale (MIES)), while connectivity between this region and the precuneus was associated positively with PTSD symptoms but negatively with total MI scores. Fulton et al. (62) extended these findings to civilians, using the Moral Injury Exposure and Symptom Scale for Civilians (MIESS-C), showing that betrayal-related MI and rumination were associated with elevated low-frequency activity in the precuneus and medial prefrontal cortex—consistent with a role for DMN-mediated self-focused cognition.

Task-based fMRI research, particularly using script-driven memory recall of PMIEs (versus neutral targets), has shown that recalling morally injurious events engages networks supporting emotion, salience, and self-processing. Lloyd et al. (63) reported greater salience-network, a brain system that underlies processing sensory, visceral and arousing content, as well as regions that involve top-down control of emotion in the prefrontal cortex in military and public-safety personnel with PTSD recalling PMIEs. This is consistent with greater involvement of neural systems associated with bodily sensation as well as regulatory control during the recollection of PMIEs. Terpou et al. (64) found stronger DMN (precuneus) connectivity in civilian-related PTSD compared to military/ law-enforcement PTSD, reinforcing a role for DMN-mediated self-processing in trauma. Kearney et al. (65) showed altered connectivity—often hyperconnectivity—between the sensorimotor network (SMN) and posterior DMN in PTSD, arguing that failures to integrate sensorimotor traces into self-referential memory may underlie MI-related disruptions. Andrews et al. (66) found that direct gaze during PMIE recall increased right temporoparietal-junction activation in PTSD, consistent with increased recruitment of regions often associated with theory-of-mind and self-referential processing. Other task-based paradigms provide converging evidence. Fulton et al. (67) found that MI scores correlated with amygdala–somatosensory connectivity during an affective Stroop task, moderated by race, consistent with the possibility that MI may involve emotion–body integration processes shaped by lived experience (e.g., racial trauma). Most recently, our own work (68) shows that MI-themed reasoning content disrupts analytic processing and engages the right posterior parahippocampus, indicating that MI-related material may engage contextual memory associations that are associated with disruptions in analytic reasoning.

Taken together, these findings suggest that DMN, salience, and sensorimotor networks—as well as regions supporting autobiographical memory, emotion, and theory of mind—may play an important role in MI. They are consistent with the possibility of converging mechanisms involving self-related processing, bodily/emotional salience, and challenges integrating morally relevant experiences into autobiographical memory. Outstanding questions concern causal mechanisms, developmental pathways, and how MI-related neural patterns interact with clinical symptoms and resilience. Again, it is important to reemphasize that due to the correlational nature of neuroimaging findings, any observed associations between activation in brain regions (and systems) and outcomes of interest need to be triangulated with other sources of data (e.g., lesion and patient data, longitudinal data, developmental data) to support causal inferences. In other words, although this approach can be used to identify processes and mechanisms associated with outcomes of interest, in isolation it does not allow for causal inferences.

## 3 Discussion

This paper summarizes a multi-method approach to better understanding the moral domain of military operations. While these findings are promising, several limitations should be noted. The synthesis draws heavily on our own research program, raising the possibility of confirmation bias, and relies on specific samples and self-report measures that may limit generalizability and introduce response biases. Similarly, not all methods used contribute equal inferential weight: large-scale surveys offer broader generalizability but rely on retrospective self-reports, experimental manipulations support stronger causal inference but may lack realism, and high-fidelity training studies provide greater ecological validity while limiting experimental control. Finally, as in the wider field, publication bias may favor positive or theory-consistent findings. Although these trade-offs complicate interpretation, they also represent a strength insofar as converging evidence across methods can increase confidence in underlying patterns. Together, these considerations suggest that conclusions should be interpreted cautiously while recognizing the value of integrating complementary methodological approaches.

**Figure 1:**
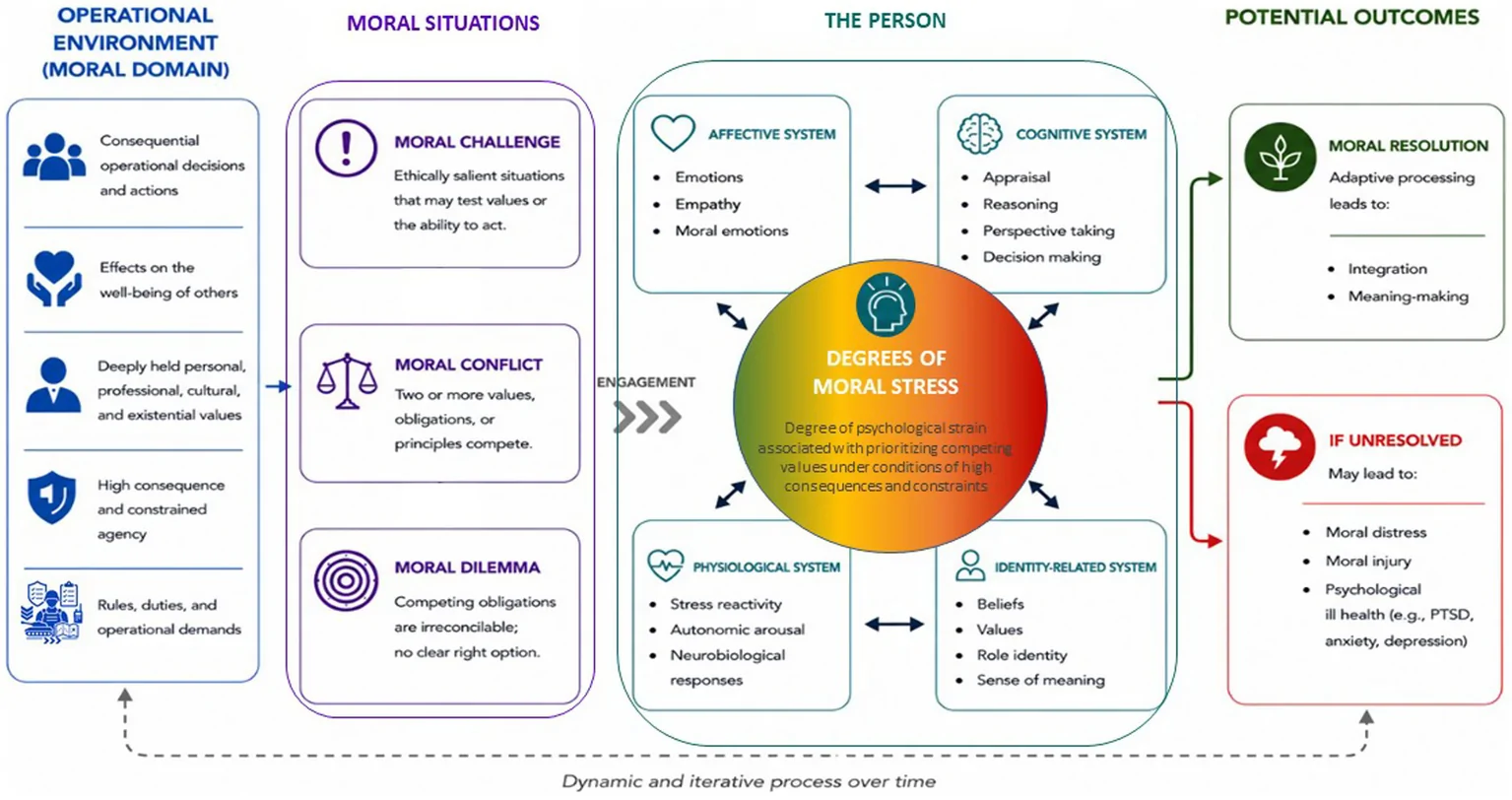
Framework depicting the emergence of moral challenges and stress in military operations. Morally salient operational situations may generate moral challenges, conflicts or moral dilemmas, engaging affective, cognitive, physiological and identity-related processes. Resulting moral stress may be resolved through integration and meaning making or, if unresolved, contribute to moral distress, moral injury and/or other adverse psychological and existential outcomes.

Drawing on the integrated findings of our research program, Figure 1 presents a preliminary framework that conceptualizes the moral domain of military operations, that is, those operational decisions and actions that have consequential effects on the well-being of others and implicate deeply held personal, professional, cultural, and existential values. These operational environments can give rise to moral challenges—ethically salient situations that may test values or the ability to act—which can include moral conflicts, where values compete, and in some cases moral dilemmas, where competing obligations are irreconcilable. Engagement with these situations involves interacting affective, cognitive, physiological, and identity-related systems and may generate moral stress, defined as psychological strain associated with prioritizing competing values under conditions of high consequence and constrained agency, even in the absence of wrongdoing. These processes may lead either to moral resolution, characterized by integration and meaning-making, or, if unresolved, to moral distress, moral injury, and related negative psychological outcomes like PTSD, anxiety and depression. Emerging methods, including virtual reality and neuroimaging, offer potential for more precise and ecologically grounded investigation of these processes.

Looking to the future, this perspective also begins to suggest the potential importance of identifying morally demanding situations in advance, rather than focusing solely on post-event outcomes, and supports continued efforts to integrate moral considerations into training, leadership, and organizational support—not only to support moral decision making and operational effectiveness, but also to enhance overall psychological resilience. Although developed within a military context, these insights may well be relevant to first responder occupations, suggesting potential avenues for supporting psychological resilience. These include increased acknowledgment of stress associated with morally charged situations; preparation for morally demanding experiences; organizational recognition of moral burden; and leadership- and organization-supported opportunities for meaning-making and moral resolution.

## Data Availability

The datasets analyzed in this study are not publicly available because they contain sensitive personal information and are subject to ethical and confidentiality restrictions. To protect participant privacy and comply with the conditions under which the data were collected, the original datasets cannot be shared or made available to third parties.

## Footnotes

2 Service members temporarily assigned—individually or in a small group—to another unit, headquarters, deployment or mission to provide specific expertise or additional capacity, rather than with their parent unit.

3 We use the more conservative term of moral conflict (vs dilemma), as it refers to a broader category covers all situations in which contradictory moral values and or obligations are apparent, some of these will reach the level of a moral dilemma.
